# Use of Breast Cancer Risk Factors to Identify Risk-Adapted Starting Age of Screening in China

**DOI:** 10.1001/jamanetworkopen.2022.41441

**Published:** 2022-11-10

**Authors:** Yadi Zheng, Xuesi Dong, Jiang Li, Chao Qin, Yongjie Xu, Fei Wang, Wei Cao, Changfa Xia, Yiwen Yu, Liang Zhao, Zheng Wu, Zilin Luo, Wanqing Chen, Ni Li, Jie He

**Affiliations:** 1 Office of Cancer Screening, National Cancer Center/National Clinical Research Center for Cancer/Cancer Hospital, Chinese Academy of Medical Sciences and Peking Union Medical College, Beijing, China; 2 Jiangsu Key Lab of Cancer Biomarkers, Prevention and Treatment, Jiangsu Collaborative Innovation Center for Cancer Personalized Medicine, Nanjing Medical University, Nanjing, China; 3 Department of Thoracic Surgery, National Cancer Center/National Clinical Research Center for Cancer/Cancer Hospital, Chinese Academy of Medical Sciences and Peking Union Medical College, Beijing, China

## Abstract

**Importance:**

Although current guidelines highlight the need for earlier screening in women at increased risk of breast cancer in China, data on risk-adapted starting ages of screening are limited.

**Objective:**

To explore the risk-adapted starting age of breast cancer screening in China, with comprehensive consideration of breast cancer risk factors.

**Design, Setting, and Participants:**

A multicenter community-based cohort study was conducted under the framework of the Cancer Screening Program in Urban China. Data were collected from January 1, 2013, to December 31, 2018, for unscreened community-dwelling women aged 40 to 74 years without a history of cancer, kidney dysfunction, or severe heart, brain, or lung disease. Data analysis was performed from October 1, 2021, to August 16, 2022.

**Exposures:**

Baseline characteristics associated with breast cancer, including first-degree family history of breast cancer, benign breast disease, breastfeeding, age at menarche, and body mass index.

**Main Outcomes and Measures:**

Outcomes included breast cancer diagnosis and age at diagnosis. Risk-adapted starting age of screening was defined as the age at which women with different levels of breast cancer risk attained a 10-year cumulative risk level similar to women aged 50 years in the general population.

**Results:**

Of the 1 549 988 women enrolled in this study, 3895 had breast cancer (median follow-up, 4.47 [IQR, 3.16-6.35] years). Participants were divided into different risk groups according to breast cancer risk scores (driven by risk factors including first-degree family history of breast cancer, benign breast disease, breastfeeding, age at menarche, and body mass index). Using the 10-year cumulative risk of breast cancer at age 50 years in the general population as a benchmark (2.65% [95% CI, 2.50%-2.76%]), the optimal starting age of screening for women with high, medium, or low risk of breast cancer was identified as 43, 48, or after 55 years, respectively. An online calculator was developed to calculate an individual’s optimal starting age of screening.

**Conclusions and Relevance:**

This study identifies the risk-adapted starting age of breast cancer screening based on the principle of equal management of equal risks, which may inform updates of current screening guidelines.

## Key Points

**Question:**

At what age should women with varying levels of breast cancer risk start screening?

**Findings:**

In this cohort study of 1 549 988 women in China, participants were divided into groups according to breast cancer risk scores (driven by risk factors including first-degree family history of breast cancer, benign breast disease, breastfeeding, age at menarche, and body mass index). Using the 10-year cumulative risk of breast cancer at age 50 years in the general population as a benchmark, the optimal starting age of screening was identified as 43, 48, or after 55 years for women with high, medium, or low risk, respectively.

**Meaning:**

These results identify the risk-adapted starting age at which to begin screening in women with varying levels of breast cancer risk in China, which may inform updates of current screening guidelines.

## Teaser

This cohort study explores the use of a risk-adapted starting age for breast cancer screening in China, considering breast cancer risk factors in community-dwelling women aged 40 to 74 years with different risk levels.

## Introduction

Breast cancer in female individuals is the leading cause of cancer incidence and the fifth leading cause of cancer mortality worldwide, accounting for 11.7% of new cancer cases and 6.9% of cancer deaths in 2020. Among women, breast cancer ranks first for cancer incidence and mortality in most countries.^1^ From 2000 to 2016, there was a significant increase in age-standardized breast cancer incidence rates among female individuals and an upward trend in breast cancer mortality rates in China.^2^

Evidence has consistently shown that screening and early detection reduce breast cancer mortality.^3^ In theory, screening interventions should be applied to high-risk populations based on a risk-adapted screening strategy^4,5^; however, the evidence is limited worldwide.^6^ To our knowledge, most randomized clinical trials and guidelines for breast cancer screening have recommended a one-size-fits-all approach in which all women are advised to begin screening at age 50 years, even those with risk factors (eg, a family history of breast cancer).^3,7,8,9,10,11^ A one-size-fits-all or age-oriented screening strategy does not consider individual variation in terms of breast cancer risk,^12^ which is not beneficial for young women at increased risk whose breast cancer incidence rate has gradually increased with distant-stage diseases in recent years.^13,14^ Therefore, a risk-adapted starting age of screening is warranted to ensure the fairness and effectiveness of breast cancer screening applications.

The recommended initial age for breast cancer screening in most guidelines is 50 years, which is consistent with the peak age of breast cancer diagnosis in China.^15^ To explore the risk-adapted starting age of breast cancer screening in China, we used data from the Cancer Screening Program in Urban China (CanSPUC)—the largest nationwide multicenter community-based screening cohort—and treated 10-year cumulative risk at age 50 years as the benchmark. We hypothesized that women with different levels of breast cancer risk should be screened at a risk-adapted age.

## Methods

This cohort study was approved by the ethics committees of the China National Cancer Center/Cancer Hospital, the Chinese Academy of Medical Sciences, and Peking Union Medical College and of all hospitals involved. All participants provided written informed consent. The study followed the Strengthening the Reporting of Observational Studies in Epidemiology (STROBE) reporting guideline.

### Study Design and Participants

We performed this multicenter community-based cohort study under the framework of the CanSPUC government-supported cancer screening program initiated in October 2012. In brief, residents aged 40 to 74 years living in participating cities who did not have a history of cancer, kidney dysfunction, or severe disease of the heart (coronary atherosclerotic, congenital, rheumatic, or hypertensive heart disease), brain (ischemic or hemorrhagic cerebrovascular disease), or lungs (chronic obstructive pulmonary disease, pneumoconiosis, or silicosis) were recruited through telephone calls, personal encounters, social media, and community advertisements. Participants answered a comprehensive questionnaire about their exposure to risk factors, and women with increased breast cancer risk were recommended to undergo ultrasound or mammography screening. Unscreened participants who entered the cohort from January 1, 2013, to December 31, 2018, were analyzed for this study, which covered a total of 19 provinces in China.

### Covariates, Outcomes, and Follow-up

The baseline survey assessed the following covariates: age (40-44, 45-49, 50-54, 55-59, 60-64, 65-69, or 70-74 years), marital status (married or unmarried, divorced, or widowed), body mass index (BMI, calculated as weight in kilograms divided by height in meters squared; <18.5, 18.5-23.9, 24-27.9, or ≥28),^16^ smoking status (nonsmoker or smoker), drinking status (nondrinker or drinker), age at menarche (<13 or ≥13 years),^9^ delivery history (yes or no), breastfeeding (yes or no), benign breast disease (yes or no), and first-degree family history of breast cancer (yes or no). Smokers were defined as those who were currently smoking or had previously smoked tobacco more than once per day for at least 6 months. Drinkers were defined as those who were currently drinking or had previously consumed alcohol more than once a week for at least 6 months. Delivery history included natural labor and cesarean birth. Benign breast disease included hyperplasia of mammary glands, nodules, ductal ectasia, or benign fibroma.

Study outcomes included breast cancer diagnosis and age at diagnosis. *International Statistical Classification of Diseases, Tenth Revision* codes were used to identify women who developed breast cancer as the primary tumor, in which breast cancer was coded as C50.

All cancer-related examinations and results were obtained from hospital medical records, the China National Central Cancer Registry, and the health insurance system. Women were followed up from the cohort entry date and left the study at the date of breast cancer diagnosis, death, emigration, or study end (August 20, 2021), whichever came first.

### Statistical Analysis

Continuous variables are expressed as the mean (SD), and the *t* or Wilcoxon rank-sum test was used to compare differences between groups. Categorical variables are expressed as percentages, and the χ^2^ or Fisher exact test was used to compare differences between groups. Breast cancer risk factors were identified with Cox proportional hazards regression models and clinical significance. The multivariable Cox proportional hazards regression model including breast cancer risk factors was used to calculate the risk score for each participant. Risk division was performed according to risk score. Risk scores were divided into different risk levels according to the number of participants and the trends in 10-year cumulative risk curves.

Ten-year cumulative risks were calculated as follows.^17^ The age-specific annual incidence rate equaled the number of cases for each age divided by person-years for that age. The 10-year cumulative incidence rate was calculated as the sum of each subsequent 10-year age-specific annual incidence rate at each age. Finally, 10-year cumulative risk equaled 1 – exp (– the 10-year cumulative incidence rate). The risk-adapted starting age of screening was defined as the age at which women with a particular risk of breast cancer attained a similar level of 10-year cumulative risk to the general population at age 50 years.

A 2-fold cross-validation method^18^ for internal validation of 10-year cumulative risk for different risk levels was conducted to assess the stability of the model used to calculate risk scores. Data analysis was performed from October 1, 2021, to August 16, 2022. In China, current guidelines recommend that women at average risk receive breast cancer screening starting at age 45 years, although this is based on expert opinion and lacks population-based evidence.^19^ Therefore, 10-year cumulative risk at age 45 years was also treated as the alternative benchmark for sensitivity analysis.

All analyses were conducted using R version 4.1.2 (R Foundation for Statistical Computing). All statistical tests were 2 sided, and *P* < .05 was considered statistically significant.

## Results

### Population Characteristics

**Figure 1.**
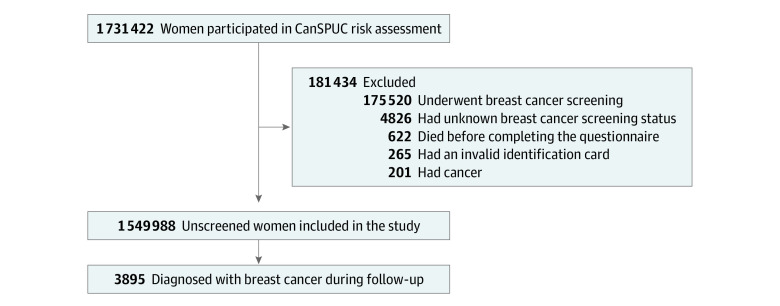
Study Population and Exclusion Criteria CanSPUC indicates Cancer Screening Program in Urban China.

Of the 1 731 422 women in the CanSPUC cohort, 1 549 988 were included in this study (Figure 1). There were 3895 patients with incident breast cancer during follow-up (median follow-up, 4.47 [IQR, 3.16-6.35] years). Baseline characteristics are presented in Table 1. Patients with incident breast cancer were typically older (aged 50-54 years: 18.7% vs 17.8%; 55-59 years: 19.5% vs 15.9%; and 60-64 years: 20.1% vs 17.5%; *P* < .01) and had a higher BMI (24-27.9: 37.7% vs 35.9%; and ≥28: 10.7% vs 9.5%; *P* < .01), younger age at menarche (12.5% vs 10.1%; *P* < .01), no breastfeeding history (9.9% vs 7.4%; *P* < .01), benign breast disease (28.2% vs 23.1%; *P* < .01), and a first-degree family history of breast cancer (9.0% vs 6.9%; *P* < .01). For women in the general population, the risk of developing breast cancer in the next 10 years was 2.65% (95% CI, 2.50%-2.76%) at age 50 and 2.32% (95% CI, 2.19%-2.44%) at age 45.

**Table 1.** Baseline Characteristics of Unscreened Women in the Cancer Screening Program in Urban China Cohort

Characteristics	No. of participants (%)			HR (95% CI)
	With breast cancer (n = 3895)^a^	Without breast cancer (n = 1 546 093)^b^	Total (N = 1 549 988)^c^	
Age, y				
40-44	472 (12.1)	228 024 (14.7)	228 496 (14.7)	1 [Reference]
45-49	594 (15.3)	249 590 (16.1)	250 184 (16.1)	1.17 (1.04-1.32)
50-54	730 (18.7)	274 458 (17.8)	275 188 (17.8)	1.31 (1.17-1.47)
55-59	761 (19.5)	246 516 (15.9)	247 277 (16.0)	1.51 (1.34-1.70)
60-64	781 (20.1)	269 877 (17.5)	270 658 (17.5)	1.45 (1.29-1.62)
65-69	467 (12.0)	214 112 (13.8)	214 579 (13.8)	1.16 (1.02-1.31)
70-74	90 (2.3)	63 516 (4.1)	63 606 (4.1)	1.01 (0.99-1.27)
Marital status				
Married	3687 (94.7)	1 467 043 (94.9)	1 470 730 (94.9)	1 [Reference]
Unmarried, divorced, or widowed	208 (5.3)	79 030 (5.1)	79 238 (5.1)	1.06 (0.92-1.22)
BMI				
<18.5	54 (1.4)	31 631 (2.1)	31 685 (2.0)	0.71 (0.54-0.93)
18.5-23.9	1952 (50.2)	809 954 (52.5)	811 906 (52.5)	1 [Reference]
24-27.9	1464 (37.7)	554 150 (35.9)	555 614 (35.9)	1.11 (1.04-1.19)
≥28	416 (10.7)	146 059 (9.5)	146 475 (9.5)	1.23 (1.11-1.37)
Smoking status				
Nonsmoker	3745 (96.1)	1 491 480 (96.5)	1 495 225 (96.5)	1 [Reference]
Smoker	150 (3.9)	54 582 (3.5)	54 732 (3.5)	1.11 (0.95-1.31)
Drinking status				
Nondrinker	3544 (91.0)	1 412 029 (91.3)	1 415 573 (91.3)	1 [Reference]
Drinker	351 (9.0)	134 002 (8.7)	134 353 (8.7)	1.06 (0.95-1.18)
Age at menarche, y				
≥13	3406 (87.5)	1 387 635 (89.9)	1 391 041 (89.9)	1 [Reference]
<13	485 (12.5)	156 392 (10.1)	156 877 (10.1)	1.29 (1.17-1.42)
Delivery history				
No	87 (2.2)	35 183 (2.3)	35 270 (2.3)	1 [Reference]
Yes	3807 (97.8)	1 510 729 (97.7)	1 514 536 (97.7)	0.97 (0.79-1.20)
Breastfeeding				
No	377 (9.9)	112 315 (7.4)	112 692 (7.4)	1 [Reference]
Yes	3445 (90.1)	1 402 974 (92.6)	1 406 419 (92.6)	0.74 (0.66-0.82)
Benign breast disease				
No	2797 (71.8)	1 188 348 (76.9)	1 191 145 (76.9)	1 [Reference]
Yes	1097 (28.2)	357 562 (23.1)	358 659 (23.1)	1.33 (1.24-1.43)
First-degree family history of breast cancer				
No	3544 (91.0)	1 438 695 (93.1)	1 442 239 (93.1)	1 [Reference]
Yes	351 (9.0)	107 342 (6.9)	107 693 (6.9)	1.37 (1.22-1.52)

Abbreviations: BMI, body mass index; HR, hazard ratio.

^a^ Data were missing for BMI (calculated as weight in kilograms divided by height in meters squared) for 9 participants, age at menarche for 4, delivery history for 1, breastfeeding for 73, and benign breast disease for 1.

^b^ Data were missing for marital status for 20 participants, BMI for 4299, smoking status for 31, drinking status for 62, age at menarche for 2066, delivery history for 181, breastfeeding for 30 804, benign breast disease for 183, and first-degree family history of breast cancer for 56.

^c^ Data were missing for marital status for 20 participants, BMI for 4308, smoking status for 31, drinking status for 62, age at menarche for 2070, delivery history for 182, breastfeeding for 30 877, benign breast disease for 184, and first-degree family history of breast cancer for 56.

### Risk Factors and Risk Scores for Breast Cancer Incidence

All selected risk factors (eg, first-degree family history of breast cancer, benign breast disease, breastfeeding, age at menarche, and BMI) used to calculate breast cancer risk scores were significantly associated with breast cancer incidence in the multivariate Cox regression model. Hazard ratios (HRs) ranged from 0.70 (95% CI, 0.53-0.92) to 1.27 (95% CI, 1.15-1.40; eTable 1 in the Supplement).

The multivariable Cox proportional hazards regression model was used to calculate the risk score for each participant as follows:

0.15190 × first-degree family history of breast cancer + 0.22453 × benign breast disease – 0.25229 × breastfeeding + 0.24038 × age at menarche younger than 13 years – 0.35994 × BMI <18.5 + 0.10798 × BMI 24-27.9 + 0.22317 × BMI ≥28.

**Figure 2.**
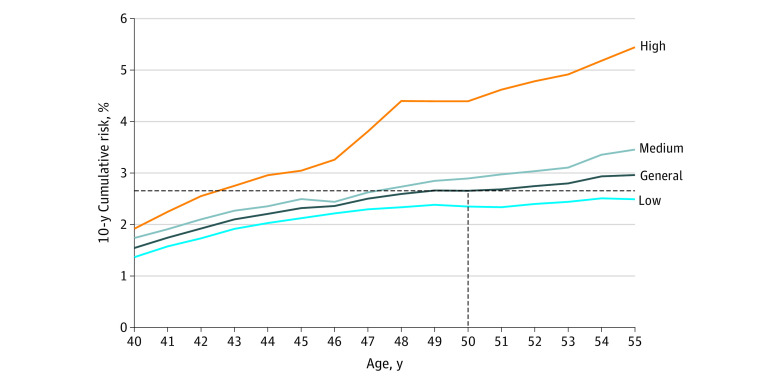
Age-Specific 10-Year Cumulative Risk of Breast Cancer for Women With Low, Medium, or High Risk

Risk scores were first divided into 10 levels from low to high, then classified into 5 risk levels due to the small number of participants in the lower- and higher-level groups (eFigure 1 in the Supplement). Because of similar trends in the 10-year cumulative risk curves, we further classified risk scores into 3 levels (low, medium, or high) (Figure 2).

Compared with women in the low-risk group, those in the medium-risk (HR, 1.32 [95% CI, 1.24-1.41]; *P* < .001) and high-risk (HR, 1.75 [95% CI, 1.53-1.99]; *P* < .001) groups had a greater tendency to develop breast cancer (Table 2). The mean (SD) age of diagnosed breast cancer in the general population was 58 (8) years, which was significantly older than that for the high-risk group (57 [7] years; *P* < .001) and similar to that for the low-risk (59 [8]; *P* = .07) and medium-risk (58 [8] years; *P* = .28) groups (eTable 2 in the Supplement).

**Table 2.** Risk-Adapted Starting Age of Breast Cancer Screening in Women With Different Risk Levels

Population	No. of participants		HR (95% CI)	Risk-adapted starting age of mass screening, y^a^	
	With breast cancer	Without breast cancer		Per He et al^19^	Per Nelson et al^3^
General	3895	1 546 093	NA	45	50
Risk					
Low	2001	902 087	1 [Reference]	48	>55
Medium	1650	578 730	1.32 (1.24-1.41)	44	48
High	244	65 276	1.75 (1.53-1.99)	42	43

Abbreviations: HR, hazard ratio; NA, not applicable.

^a^ Other starting ages of screening include the evidence-based, risk-adapted ages recommended in this study.

### Evaluation of Risk-Adapted Starting Age of Breast Cancer Screening

Women with low, medium, or high breast cancer risk (aged >55, 48, or 43 years, respectively) reached 10-year cumulative breast cancer risk equal to that of women with average risk at age 50 years in the general population (Figure 2 and Table 2). Comparable results were found in the 2-fold cross-validation analysis. As a result, 10-year cumulative risk curves for women with different risk levels in both the development and validation data sets were analogous across age (eFigure 2 in the Supplement). We also developed an online tool to calculate an individual’s starting age of breast cancer screening.^20^

### Sensitivity Analysis

Using 10-year cumulative risk at age 45 years as an alternative risk benchmark, our proposed breast cancer risk stratification demonstrated consistent breast cancer incidence trends in unscreened women in the CanSPUC cohort. As a result, risk equal to that of women at average risk at age 45 years in the general population was achieved 3 years later in the low-risk group and 1 and 3 years earlier in the medium-risk and high-risk groups, respectively (Table 2 and eFigure 3 in the Supplement).

## Discussion

This cohort study examined the risk-adapted starting age of breast cancer screening in China by using CanSPUC population-based national cancer screening program data, with more than 1 million women eligible. Women with low breast cancer risk were proposed to start screening after age 55 years, whereas those with medium or high risk were proposed to begin screening at age 48 or 43 years, respectively. We also generated an online tool (http://cancerrc.ncsis.org.cn/#/utilshome) to calculate an individual’s optimal starting age and to support decision-making in breast cancer screening.

In this study, breast cancer risk factors used for risk division included first-degree family history of breast cancer, benign breast disease, breastfeeding, age at menarche, and BMI. A family history of breast cancer is a well-demonstrated risk factor^10,21,22^ and remains a major factor for screening and prevention counseling. Individuals with benign breast disease are also reported to have a higher risk of breast cancer.^23,24^ A meta-analysis^7^ showed that Chinese women with benign breast disease had a 2.68 times greater risk of breast cancer (OR, 2.68 [95% CI, 2.06-3.49]) than those without the disease. Evidence suggests that breastfeeding is inversely associated with breast cancer^25^ and exerts a notable protective effect against hormone receptor-negative breast cancers.^26^ Younger age at menarche is also associated with a higher risk of breast cancer. A meta-analysis of 117 studies showed a 5.0% reduction in breast cancer risk for every year older at menarche.^9^ Furthermore, the results of a meta-analysis of studies in East Asian women showed that obesity is independently associated with increased risk of breast cancer in postmenopausal women.^27^ Although all of these factors were considered in the China Guideline for the Screening and Early Detection of Female Breast Cancer,^19^ the recommended starting age of screening for women at average and increased risk was based on expert opinion and lacked population-based evidence in Chinese women. Evidence of a risk-adapted starting age of screening is needed to determine populations at high risk and promote screening of these individuals, which can further aid in efficient allocation of limited medical resources, especially in developing countries such as China.

To date, studies focused on risk-adapted starting ages of breast cancer screening have been conducted with the Swedish family cancer data sets by Mukama et al.^11,18,28^ These authors considered a family history of breast cancer, reproductive profile (eg, age at first live birth and parity), and other family history of cancers separately in their study, which makes it difficult for individuals with more than 1 category of risk factors to decide when to screen.

Several improvements were made in our study. First, we analyzed more than 1 million community-dwelling women from a multicenter, population-based, prospective cohort. Baseline characteristics in our study (eg, age and marital status) were also similar to data from the 2020 China Population Census Yearbook,^29^ which also means that the population analyzed here is representative of Chinese women in the general population. Second, all cancer-related examinations and results were obtained from hospital medical records, the China National Central Cancer Registry, and the health insurance system, which contributed to study reliability. Third, we combined 5 factors associated with breast cancer and classified women into groups with different risk levels according to combined risk scores, which is more practical for clinical use. In addition, although the median follow-up time of the study was no more than 5 years, we found that the incident age of breast cancer in women in the general Chinese population was significantly older compared with women at high risk, which supports the recommendation of a risk-adapted starting age of breast cancer screening. We believe that a difference would also be found between the medium-risk or low-risk groups and the general population of women with longer follow-up. Finally, we generated an online calculator based on the proposed risk-adapted age to support individual decision-making.

### Limitations

This study has some limitations. First, the age of 40 years recommended by certain guidelines was not explored due to the limitation of the starting age in this study.^30,31^ Second, although 5 factors were selected for risk division in our study according to clinical and statistical significance, more key variables associated with breast cancer (eg, mammographic density^32,33^) should be collected and used for risk division in future research. In addition, risk-adapted starting age in our study was defined as the age at which women with different risk levels attained a 10-year cumulative risk comparable to the average risk for women at the recommended age in the general population. The cost-effectiveness of this approach was not taken into consideration and should be assessed in further studies. In addition, external validation should also be conducted. Screening methods such as mammography^34^ and clinical breast examination^35^ are proven to be effective for breast cancer screening. Screening strategies include not only the starting age of screening but also screening methods and screening intervals, which should be investigated for risk-adapted breast cancer screening in the future to detect women at high risk for breast cancer. Doing so helps to focus limited resources on these individuals, especially in developing countries such as China.

## Conclusions

This cohort study reports the risk-adapted starting age of breast cancer screening in China. In addition, we developed a website to calculate an individual’s optimal starting age of screening, which may support the decision-making process and inform the update of current breast cancer screening guidelines. Our findings contribute to the principle of equal management of equal risks in breast cancer prevention and may help narrow the focus to women at high risk. Doing so may optimize the allocation of limited screening resources, especially in developing countries like China. Future research on risk-adapted screening methods and screening intervals is needed.

## Notes

Supplement. **eFigure 1.** Age-Specific 10-Year Cumulative Breast Cancer Risk for Women With 5 Risk Score Levels, Using Age 50 Years for Women in the General Population as a Benchmark **eFigure 2.** Age-Specific 10-Year Cumulative Breast Cancer Risk for Women With 3 Risk Score Levels in the Development and Validation Data Sets **eFigure 3.** Age-Specific 10-Year Cumulative Breast Cancer Risk for Women With 3 Risk Score Levels, Using Age 45 Years for Women in the General Population as a Benchmark **eTable 1.** Association of Risk Factors and Breast Cancer Incidence **eTable 2.** Incident Age of Breast Cancer in Women With Different Risk Levels
