# Supplementary material for: Use of Breast Cancer Risk Factors to Identify Risk-Adapted Starting Age of Screening in China
Source: JAMA Netw Open. 2022 Nov 10;5(11):e2241441. doi: 10.1001/jamanetworkopen.2022.41441 (PMC9650608; doi:10.1001/jamanetworkopen.2022.41441)
Supplement: Supplement. — eFigure 1. Age-Specific 10-Year Cumulative Breast Cancer Risk for Women With 5 Risk Score Levels, Using Age 50 Years for Women in the General Population as a Benchmark eFigure 2. Age-Specific 10-Year Cumulative Breast Cancer Risk for Women With 3 Risk Score Levels in the Development and Validation Data Sets eFigure 3. Age-Specific 10-Year Cumulative Breast Cancer Risk for Women With 3 Risk Score Levels, Using Age 45 Years for Women in the General Population as a Benchmark eTable 1. Association of Risk Factors and Breast Cancer Incidence eTable 2. Incident Age of Breast Cancer in Women With Different Risk Levels [file jamanetwopen-e2241441-s001.pdf]

Supplementary Online Content

Zheng Y, Dong X, Li J, et al. Use of breast cancer risk factors to identify risk-adapted starting age of screening in China. *JAMA Netw Open*. 2022;5(11):e2241441. doi:10.1001/jamanetworkopen.2022.41441

eFigure 1. Age-Specific 10-Year Cumulative Breast Cancer Risk for Women With 5 Risk Score Levels, Using Age 50 Years for Women in the General Population as a Benchmark
eFigure 2. Age-Specific 10-Year Cumulative Breast Cancer Risk for Women With 3 Risk Score Levels in the Development and Validation Data Sets
eFigure 3. Age-Specific 10-Year Cumulative Breast Cancer Risk for Women With 3 Risk Score Levels, Using Age 45 Years for Women in the General Population as a Benchmark
eTable 1. Association of Risk Factors and Breast Cancer Incidence
eTable 2. Incident Age of Breast Cancer in Women With Different Risk Levels

This supplementary material has been provided by the authors to give readers additional information about their work.

© 2022 Zheng Y et al. *JAMA Network Open*.

eFigure 1. Age-Specific 10-Year Cumulative Breast Cancer Risk for Women With 5 Risk Score Levels, Using Age 50 Years for Women in the General Population as a Benchmark

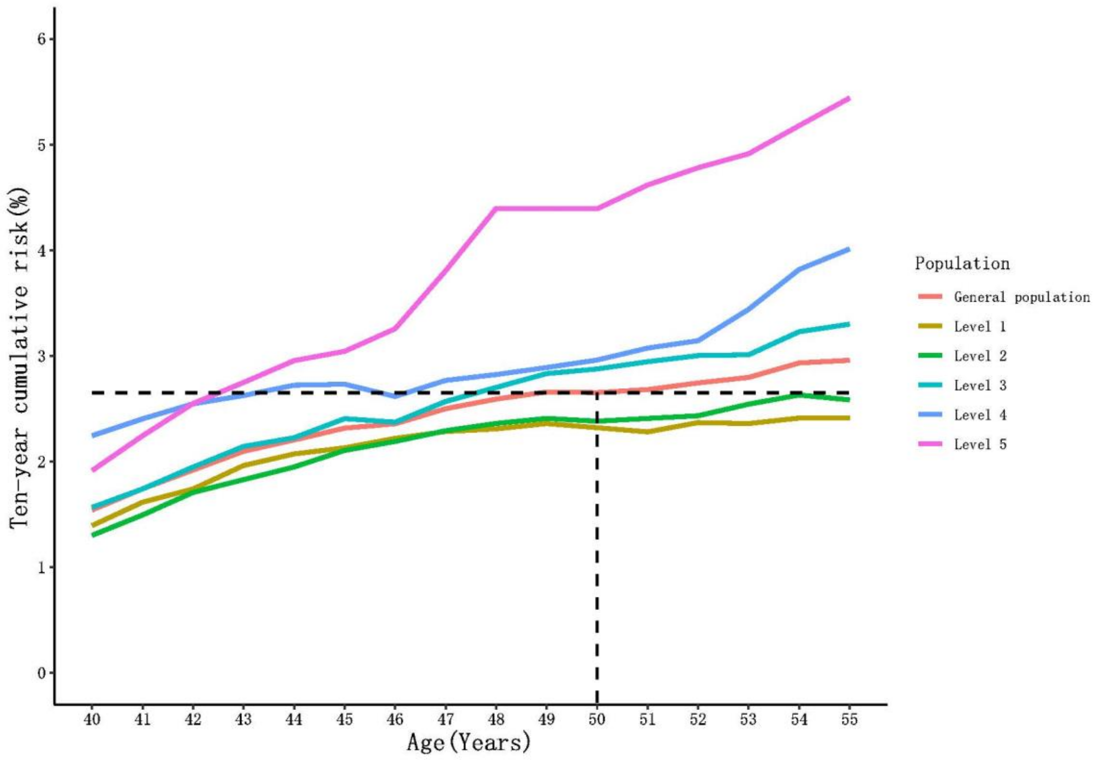

© 2022 Zheng Y et al. *JAMA Network Open*.

eFigure 2. Age-Specific 10-Year Cumulative Breast Cancer Risk for Women With 3 Risk Score Levels in the Development and Validation Data Sets

Solid line: development set; dotted line: validation set.

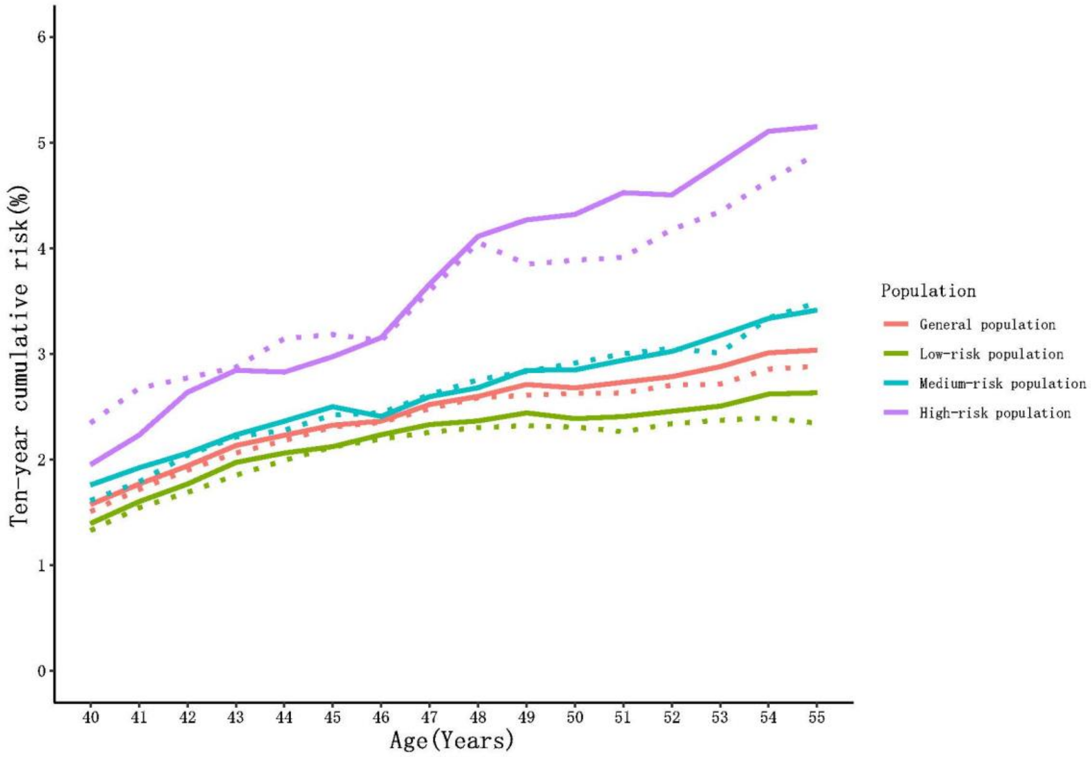

© 2022 Zheng Y et al. *JAMA Network Open*.

eFigure 3. Age-Specific 10-Year Cumulative Breast Cancer Risk for Women With 3 Risk Score Levels, Using Age 45 Years for Women in the General Population as a Benchmark

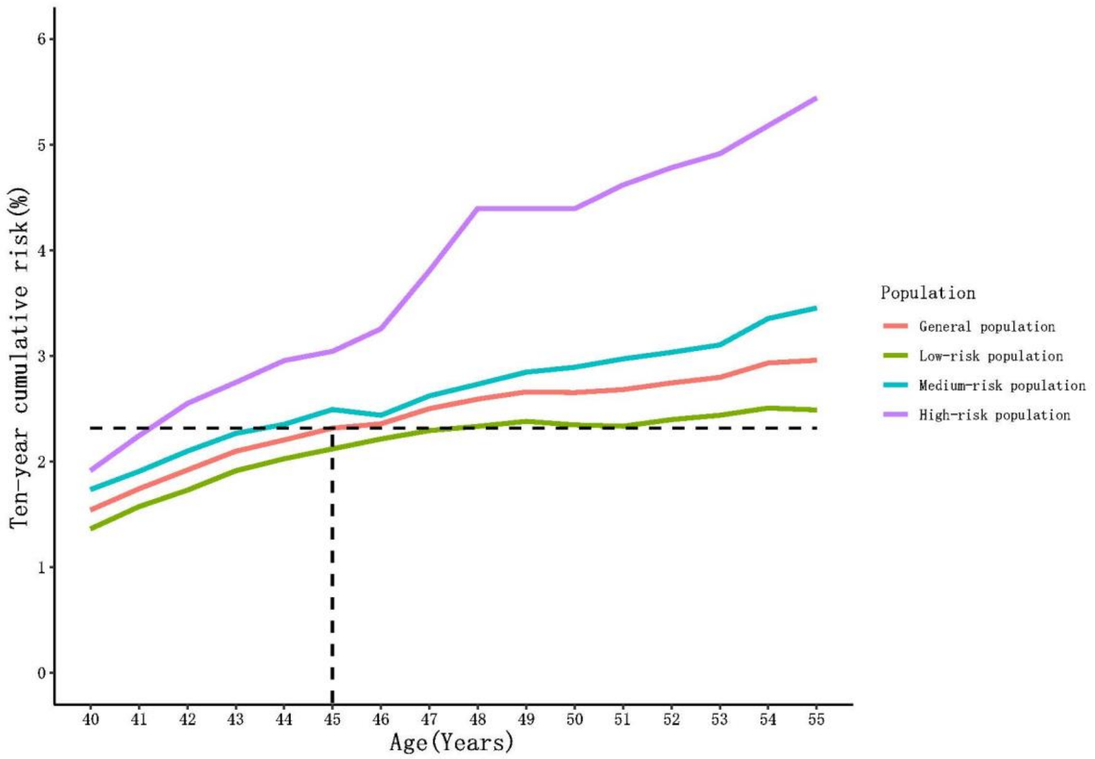

© 2022 Zheng Y et al. *JAMA Network Open*.

eTable 1. Association of Risk Factors and Breast Cancer Incidence

Variable	MV-adjusted HR (95% CI) [a]	*P* value
**First-degree family history of breast cancer**		
No	1.00 (reference)	
Yes	1.16 (1.03-1.31)	<.05
**Benign breast disease**		
No	1.00 (reference)	
Yes	1.25 (1.16-1.35)	<.001
**Breastfeeding**		
No	1.00 (reference)	
Yes	0.78 (0.70-0.87)	<.001
**Age at menarche, years**		
≥13	1.00 (reference)	
<13	1.27 (1.15-1.40)	<.001
**BMI**		
<18.5	0.70 (0.53-0.92)	<.05
18.5-23.9	1.00 (reference)	
24-27.9	1.11 (1.04-1.19)	<.01
≥28	1.25 (1.12-1.39)	<.001

BMI: Body mass index.

HR: Hazard ratio.

CI: Confidence interval.

[a] Multivariate Cox regression model.

© 2022 Zheng Y et al. *JAMA Network Open*.

eTable 2. Incident Age of Breast Cancer in Women With Different Risk Levels

Population	Follow-up period (Median (IQR), y)	Incident age of breast cancer (Mean (SD), y)	*P* value
General	4.47 (3.16-6.35)	58 (8)	
Risk			
Low	4.59 (3.18-6.38)	59 (8)	.07
Medium	4.40 (3.14-6.29)	58 (8)	.28
High	4.38 (3.12-6.19)	57 (7)	<.001

IQR: Interquartile range.

SD: Standard deviation.

© 2022 Zheng Y et al. *JAMA Network Open*.
